# Molecular links between non-alcoholic fatty liver disease and hepatocellular carcinoma

**DOI:** 10.20517/2394-5079.2019.014

**Published:** 2019-12-11

**Authors:** Sana Raza, Sangam Rajak, Baby Anjum, Rohit A. Sinha

**Affiliations:** 1 Department of Bioscience, Integral University, Lucknow 226026, India; 2 Department of Endocrinology, Sanjay Gandhi Postgraduate Institute of Medical Sciences, Lucknow 226014, India

**Keywords:** Non-alcoholic fatty liver disease, non-alcoholic steatohepatitis, hepatocellular carcinoma, Gut microbiome, dysbiosis, autophagy, ER-stress, ROS, TNFα, TLR-9, TLR-4, hyperinsulinemia

## Abstract

Non-alcoholic fatty liver disease (NAFLD) and its advanced complication, non-alcoholic steatohepatitis (NASH), have become leading causes of hepatocellular carcinoma (HCC) worldwide. In this review, we discuss the role of metabolic, gut microbial, immune and endocrine mediators which promote the progression of NAFLD to HCC. In particular, this progression involves multiple hits resulting from lipotoxicity, oxidative stress, inhibition of hepatic autophagy and inflammation. Furthermore, dysbiosis in the gut associated with obesity also promotes HCC via induction of proinflammatory cytokines and Toll like receptor signalling as well as altered bile metabolism. Additionally, compromised T-cell function and impaired hepatic hormonal action promote the development of NASH-associated HCC. Lastly, we discuss the current challenges involved in the diagnosis and treatment of NAFLD/NASH-associated HCC.

## Introduction

Hepatocellular carcinoma (HCC) is the primary form of liver cancer and is a leading cause of cancer-related mortality worldwide^[1]^. It is predominantly known to occur in patients suffering from underlying chronic liver disease and cirrhosis. Hepatitis B and C virus (HBV and HCV, respectively) infections, excessive consumption of alcohol and non-alcoholic fatty liver disease (NAFLD) historically have been recognised as the major causes of HCC; however, the incidence of virus-associated HCC is expected to decrease in the near future due to the development of effective and inexpensive vaccines for HBV and potent anti-HCV drugs^[1,2]^. In contrast, the prevalence of non-viral hepatitis continues to rise and has become the major cause for liver transplantation in Europe and the USA^[3]^. The increased prevalence of metabolic disorders, particularly diabetes, NAFLD and obesity, have led to changes in the epidemiology and aetiology of HCC^[4]^. Obesity is considered a risk factor for hepatic complications such as NAFLD and HCC^[5–9]^. Although 17%-33% of the general population is estimated to be affected by NAFLD, it reaches 75% in obese individuals and is even higher in patients with type II diabetes mellitus (T2DM)^[10,11]^. Moreover, T2DM itself is associated with an increased risk of liver damage^[12]^, including HCC^[13–15]^. Chronic damage to liver metabolism caused by alcohol and poor nutrition leads to alcoholic liver disease that can co-exist with NAFLD/non-alcoholic steatohepatistis (NASH), and thereby increases both the progression of NAFLD and the risk for NAFLD/NASH-associated HCC^[2,3]^.

This review focuses on NAFLD-associated HCC, and describes its epidemiology and the clinical, cellular, metabolic, microbiome and endocrine factors that promote the development of HCC from NAFLD. We also examine the molecular pathways that lead to progression from NAFLD to HCC as well as the challenges and future directions for its treatment and prevention.

## NAFLD increases the risk of liver cancer

NAFLD encompasses a spectrum of liver pathologies which involve an accumulation of triglycerides in the hepatocytes, hepatocyte apoptosis, liver inflammation and fibrosis termed as NASH, and, in extreme cases, it can progress to cirrhosis and HCC^[16]^. NAFLD is the most common cause of HCC across the globe^[16–28]^. Although the progression of NAFLD to HCC involves NASH and cirrhosis, the direct development of HCC from benign steatosis or non-cirrhotic NASH has also been reported^[29,30]^. The increased prevalence of the underlying liver disease in the general population has led to an increase of 9% in the annual rates of incidence of NAFLD-associated HCC^[31]^. Interestingly, HCC can progress from NASH as well as cirrhosis. In a study cohort based on 756 patients, Piscaglia *et al.* ^[32]^ reported that 46.2% of the NAFLD associated HCC cases occurred without cirrhosis. Similar results were reported by a Japanese study, in which 49% of NAFLD associated HCC cases arose without cirrhosis^[33]^, and a German study where 41.7% of the cases arose without cirrhosis^[34]^. Furthermore, in animal models, diet-induced NAFLD leads to spontaneous HCC^[35]^.

## Cellular mechanisms involved in NAFLD pathogenesis

NAFLD is a complex disease with multiple modifiers such as diet, lifestyle and gut microbiota which act in a susceptible genetic/epigenetic environment and modulate response to calorific excess^[36,37]^. The role of insulin resistance is central to this pathophysiological process and causes an increase in hepatic fat accumulation by increased deposition of free fatty acids (FFAs)^[38]^. This leads to oxidative stress, protein misfolding, autophagy inhibition and mitochondrial damage within hepatocytes, termed as “lipotoxicity”^[38]^. Chronic lipotoxicity challenges hepatocytes with both oxidative and endoplasmic reticulum (ER) stress. Oxidative stress mediated by reactive oxygen/nitrogen species (ROS/RNS) play a major role in NAFLD/NASH pathogenesis and complications. The high production of ROS causes mitochondrial damage, lipid peroxidation and low-density lipoprotein oxidation culminating into inflammation, activation of hepatic stellate cells (HSCs) leading to fibrogenesis, necrosis, cirrhosis and HCC^[39]^.

**Figure 1:**
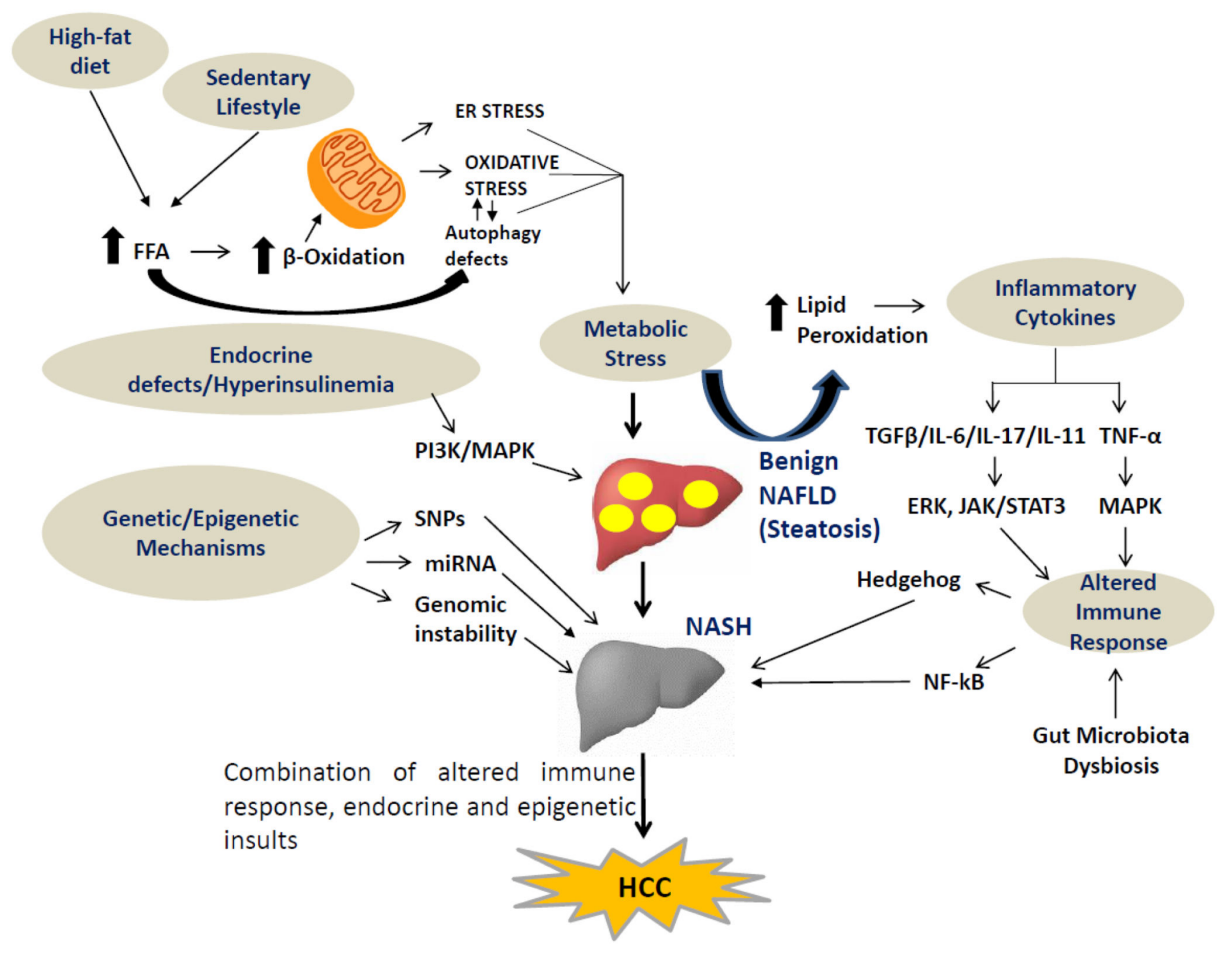
Multiple hits lead to onset and progression of NAFLD/NASH to HCC. Diverse signalling pathways involved in metabolic stress such as FFAs ER-stress, cytokine production (IL-6, IL-17, IL-11 and TGF-β), altered immune response, pro-fibrogenic mediators (hedgehog and NF-κB), gut dysbiosis and endocrine defects drive the development of NAFLD/NASH-associated HCC. NAFLD: non-alcoholic fatty liver disease; NASH: non-alcoholic steatohepatitis; HCC: hepatocellular carcinoma; FFAs: free fatty acids; ER: endoplasmic reticulum; IL-6: interleukin-6; IL-17: interleukin-17; IL-11: interleukin-11; TGF-β: transforming growth factor β; SNPs: single nucleotide polymorphisms; miRNA: micro RNA; PI3K: phosphatidylinositol 3-kinases; MAPK: mitogen-activated protein kinase; NF-κB: nuclear factor kappa-light-chain-enhancer of activated B-cells; TNF-α: tumour necrosis factor-alpha; ERK: extracellular receptor kinase; JAK: Janus kinase; STAT: signal transducer and activator of transcription

ER stress is cell activated to regulate protein synthesis and restore homeostatic equilibrium in response to accumulation of unfolded or misfolded proteins. However, deregulated or insufficient responses to ER stress in liver may lead to lipid accumulation, insulin resistance, inflammation and apoptosis, all of which play important roles in the pathogenesis of NAFLD^[40]^. These events lead to inflammation and fibrosis as macrophage infiltration, hepatic progenitor cell activation and fibrogenesis ensue^[41,42]^. There are multiple factors that contribute to the pathogenesis of NAFLD and its progression. These include: dysregulated lipid metabolism, oxidative stress, ER stress, mitochondrial dysfunction, altered immune function, and gut-microbiota imbalance acting together in a genetic/epigenetic environment [Figure 1].

## Molecular mechanisms leading to progression from NAFLD to HCC

Studies on the development of HCC suggest that carcinogenesis in hepatocytes is a consequence of genetic/epigenetic alterations as well as complex changes in energy metabolism, cell growth and proliferation and immune signalling pathways. These changes in the cells lead to inflammation, hepatocyte injury, fibrosis and progression to HCC [Figure 1].

### Genetic/Epigenetic mechanisms

Several single nucleotide polymorphisms (SNPs) have been associated with the occurrence of NAFLD and its progression to advanced fibrosis^[36]^. The patatin-like phospholipase domain-containing protein 3 (PNPLA3) gene polymorphism is associated with the progression of NASH-associated HCC^[43]^. PNPLA3 impairs triglyceride mobilisation from lipid droplets. Patients carrying the PNPLA3 polymorphism are reported to have a three-fold increased risk of developing HCC^[44,45]^. Transmembrane 6 superfamily member 2 gene (*TM6SF2*) mutations are also prevalent in NASH patients^[46]^. They are believed to be linked to liver injury in the pathogenesis of NASH-associated HCC^[47]^. Recently, membrane bound O-acetyl transferase domain containing 7 (MBOAT7) rs641738 variant associated with NAFLD progression has also been linked to HCC susceptibility^[48]^ [Figure 1].

In addition to the SNPs, genetic instability is also believed to stimulate the progression of NASH to HCC. Mutations in oncogenic genes, such as the human telomerase reverse transcriptase (*hTERT*) gene which catalyses the addition of nucleotides to the ends of eukaryotic chromosomes, tumour protein p53, cyclin dependent kinase inhibitor 2 A, albumin, catenin beta-1 and axis inhibition protein 1 (involved in Wnt/β-catenin signalling), are prevalent in exome-sequencing analysis of HCC^[49]^. Aberrant DNA methylation is also an important mechanism in NASH progression^[50]^, and can lead to silencing of genes involved in DNA repair, lipid metabolism, glucose metabolism and progression of fibrosis^[50]^. In particular, the epigenetic changes in the gene encoding chromodomain helicase DNA-binding protein 1 are reported to be linked to NASH-associated HCC^[51]^.

The expression of several microRNAs (miRNAs) also is reported to be dysregulated in many types of cancer, including NASH-associated HCC^[52]^. The miRNAs are small noncoding RNAs that down-regulate gene expression by interfering with transcription and/or translation. These miRNAs are involved in cell signalling pathways associated with oncogenesis, such as transforming growth factor (TGF)-β, Wnt/β-catenin, mitogen-activated protein kinase (MAPK) and phosphatidylinositol 3-kinases (PI3K)/AKT/mTOR pathways, which can be activated in HCC^[53]^. In particular, miRNAs known to target the inhibitors of PI3K/AKT pathway are found in HCC. In this connection, steatosis, hepatomegaly and HCC have been observed in phosphatase and tensin-deficient mice^[54]^. Several miRNAs are differentially expressed in a high fat diet mouse model during the NAFLD-NASH-HCC transitions^[55]^. Hepatic miR-340-5p, miR-484, miR-574-3p and miR-720 were expressed in NAFLD, NASH and HCC, and miR-125a-5p and miR-182 showed early and significant dysregulation during hepatic tissue damage^[55]^.

### Metabolic pathways

The association of obesity, high-fat diet and diabetes to NAFLD/NASH and its progression to HCC suggests the existence of a molecular link between energy metabolism and cell cycle control in the hepatocytes, which may be a key mechanism driving the progression of NASH to HCC. Several animal studies have been conducted to investigate NASH-associated HCC. These studies showed that the progression of NASH-associated HCC may be due to abnormal lipid metabolism, oxidative stress, ER stress and mitochondrial dysfunction acting independently or in tandem^[56,57]^ [Figure 1].

It is worth noting that mitochondrial activities such as β-oxidation, electron transfer, ATP production and ROS generation regulate the fat metabolism and energy homeostasis in hepatocytes^[58]^. During the early hepatosteatoic phase of NAFLD when there is fatty acid accumulation in hepatic cells, mitochondria prevent oxidative stress and help facilitate the partition of lipotoxic FFAs into stable triglycerides that can be stored in fat droplet, and thereby prevent oxidative stress^[59]^. However, chronic high-fat or high-fructose diet leads to lipid over-accumulation in the hepatocytes due to cellular metabolic reprogramming and accumulation of toxic metabolites^[60]^. These changes lead to imbalances in hepatic metabolism that result in excessive production of FFAs, which can cause lipotoxicity^[61]^.

The excessive accumulation of these fatty acids increases β-oxidation and ROS production, which can limit mitochondrial function^[62]^. When these mitochondrial abnormalities are accompanied by diminished intracellular antioxidant protection in NASH, pathways of fatty acid metabolism are altered^[63]^, which, in turn, can cause metabolic stress^[63]^. Overproduction of ROS frequently occurs in cancer, and is believed to play an important role in the development of HCC^[64]^. Intriguingly, oxidative stress and abnormal methylation of tumour suppressor genes are found the livers of NAFLD patients^[65]^. ER stress also contributes to hepatocyte injury and carcinogenesis in NASH^[66]^. Thus, the cross-talk among oxidative stress, ER stress and cell death pathways likely plays a role in the development of NASH and its progression to HCC^[67]^. Similar to oxidative and ER stress, autophagy dysregulation may be involved in the progression of NASH to HCC^[68]^. In this regard, impaired autophagy leads to defective lipid metabolism^[69]^, proteotoxicity^[70]^, mitochondrial dysfunction^[71]^ and inflammation^[72]^, all of which can contribute to HCC induction.

Several xenobiotic metabolising genes of the aldo-keto reductase family show parallel induction in NASH and HCC, suggesting a genetic link between NASH and its progression to HCC^[73–76]^. Thus, disturbance in hepatic cell metabolism can lead to increased cell death, DNA damage, immune cell activation and compensatory proliferation^[57]^. These changes in hepatic cells activate HSCs and induce fibrosis. If tumour surveillance and DNA damage repair are impaired in NASH, pre-malignant cells can develop, and, after critical genetic/epigenetic changes, they become clones that progress to HCC. Therefore, the cumulative effects of oxidative stress and proliferative response during inflammation and fibrosis are thought to drive the progression of HCC^[57]^.

### Gut microbiota

“Gut microbiota” refers to populations of bacteria hosted by the adult human intestine, which maintain a symbiotic relationship with the host and have a key role in the host immune system. They perform various functions in the body such as digestion of inaccessible nutrients, synthesis of vitamins and resistance to pathogens^[77]^. They are known to ferment carbohydrates such as cellulose and xylans into short-chain fatty acids (SCFAs). The liver is exposed to gut-derived products by portal circulation, which provides a defence against bacterial toxins. SCFAs improve hepatic autophagy and gut barrier function, and reduce the permeability of bacterial toxins. These gut products can reduce pro-inflammatory pathways and insulin resistance, which are associated with the progression of chronic liver disease^[78,79]^. The gut microbiota composition is dynamic and may be influenced by diet, hygiene and the use of antibiotics^[80]^. The modification of the normal microbiota termed as “dysbiosis” is believed to be associated with the progression of NAFLD and other chronic metabolic diseases^[81–85]^.

Dysbiosis in gut flora has been associated with HCC incidence in humans and animal models^[86]^. Mice kept in germ-free conditions or given antibiotics tend to develop fewer and smaller HCCs^[87,88]^. At the molecular level, dysbiosis of the gut microbiota leads to an increase in secretion of inflammatory cytokines, such as tumour necrosis factor alpha and interleukin-8 (IL-8) along with the activation of toll like receptor (TLR)-4 and TLR-9, resulting in production of IL-1β by Kupffer cells, which are star-shaped (stellate) phagocytic cells located in the liver. IL-1β promotes lipid accumulation and apoptosis in hepatocytes, causing steatosis and inflammation, as well as activation of HSCs to produce fibrogenic mediators, and accelerate HCC establishment^[89–92]^. Furthermore, dysbiosis promotes the development of NAFLD-associated HCC by modifying bile acid metabolism. Specifically, alterations in the composition of the gut microbiota can result in higher levels of deoxycholic acid and the activation of its receptor farnesoid X receptor, which provokes a senescence-associated secretory phenotype in HSCs, resulting in the secretion of various inflammatory and tumour-promoting factors in the liver, thus promoting the development of HCCs^[87,93]^ [Figure 1]. To summarise, the intestinal microbiota may promote the development of NAFLD-associated cirrhosis and HCC by increasing inflammatory cytokine secretion, activating TLR-4 and TLR-9 and modifying bile acid metabolism.

### Immunological pathways

Metabolic stress not only leads to increased ROS generation but also triggers the inflammatory responses, which are a pre-requisite for the progression of NASH-associated HCC. Insulin resistance and oxidative stress are known to stimulate nuclear factor kappa-light-chain-enhancer of activated B-cells pathway, which promotes hepatocyte survival^[94]^. ROS and the products of lipid peroxidation stimulate the release of inflammatory cytokines including tumour necrosis factor-alpha (TNF-α) and IL-6 from hepatic cells^[95]^. TNF-α is reported to promote hepatocellular carcinogenesis by activating hepatic progenitor cells^[67]^. IL-6 activates the signal transducer and activator of transcription 3, an oncogenic transcription factor that induces cell proliferation, inhibits apoptotic pathways and may be involved in the development of NASH-associated HCC^[96]^ [Figure 1]. Similarly, other cytokines such as IL-17A and IL-11 have also been implicated in NASH and NASH-associated HCC^[97–99]^.

Cellular injury activates the hedgehog signalling pathway, which is involved in repair and regeneration in the liver and replaces damaged hepatocyte. The Hedgehog signalling pathway is implicated in fibrogenic activation and hepatocellular ballooning^[100]^, features associated with the advancement of NASH. Impairment of the Hedgehog pathway also leads to dysregulation of cell repair mechanisms and promotes the malignant transformation involved in the progression of HCC^[101]^. The TGF-β signalling also mediates the progression of fibrogenesis through regulation of cell death and lipid metabolism in NASH^[102,103]^ [Figure 1].

The role of CD8+ and CD4+ T lymphocytes in hepatocyte damage and carcinogenesis has been studied in various animal models^[104]^. In mouse models and human samples, dysregulation of lipid metabolism in NAFLD causes a selective loss of intrahepatic CD4+ but not CD8+ T lymphocytes, leading to increased inflammation and accelerated development of HCCs^[105]^. Platelet cargo, platelet adhesion and platelet activation appear to be pivotal for NASH and subsequent hepatocarcinogenesis. In particular, platelet GPIbα is a mediator of hepatic immune cell trafficking and antiplatelet therapy (e.g., aspirin/clopidogrel and ticagrelor) has been demonstrated to prevent NASH and subsequent HCC development^[106]^. One recently published study used a new zebra fish model and reported that a high fat diet promotes non-resolving inflammation in the liver and enhances cancer progression^[107]^. The authors found that metformin inhibits high fat diet-induced HCC progression, by reducing inflammation and restoring tumour surveillance^[107]^.

### Endocrine pathways

Several hormones play important roles in the pathogenesis of NAFLD and its consequent progression to HCC. One of the most crucial in these is insulin resistance and hyperinsulinemia, which is an associated feature of NAFLD^[108]^. Insulin resistance and hyperinsulinemia are known to increase the expression of insulin and insulin-like growth factor-1 (IGF-1). Insulin and IGF-1 trigger signalling cascades by binding to their receptors, namely the insulin receptor and the IGF-1 receptor, to activate the PI3K and MAPK pathways, which are crucial in the pathogenesis of HCC since they induce cell proliferation and inhibit apoptosis^[109]^. In particular, the PI3K pathway mediates the progression of HCC by cyclin D1-dependent control of the cell cycle, mTOR dependent cellular growth and mouse double minute 2 homolog Mdm2/p53-dependent apoptosis^[110]^. Interestingly, there can be cross-talk between other signalling pathways and PI3K-mediated signalling; e.g., hippo signalling suppresses IRS2/Akt-mediated HCC development in rodent models^[111]^. Activation of the MAPK pathway by insulin resistance induces the expression of proto-oncogenes, c-fos, and c-jun, and promotes hepatic fibrosis and carcinogenesis by activating the Wnt/β-catenin signalling cascade^[112]^ [Figure 1].

NAFLD is also associated with increased circulating levels of leptin^[113]^. Leptin initiates intracellular signalling of pro-inflammatory cytokines such as TNF-α and IL-6 and activates JAK2/STAT, MAPK and PI3K signalling pathways by binding to its receptor in HCC cells^[114]^. Furthermore, leptins are known to upregulate hTERT expression, leading to the immortalisation of HCC cells^[115]^. NAFLD is also associated with decreased sensitivity to thyroid hormones^[116]^ and both NAFLD and HCC are associated with hypothyroidism in humans^[117,118]^. Furthermore, thyroid hormone treatment decreases hepatosteatosis and the progression of NAFLD in both rodents and humans^[119]^. Several new studies provide evidence for a potential role of androgen and the androgen receptor pathway in the development of NASH-related HCC and in the treatment of HCC^[120]^. Gender disparity exists in the incidence of NAFLD associated HCC^[121]^. Intriguingly, although males are more likely to develop both NAFLD and HCC than females, after the age of 60 this trend is reversed^[122–124]^. This has been attributed to the loss of the protective effects of oestrogen in females^[125]^. Besides hormonal stimuli, deregulation of hepatic circadian clock genes also significantly contributes towards the progression of NAFLD to HCC^[126]^.

## Future directions and conclusions

Studies from clinical and basic research have provided a better understanding of the aetiology of NASH-associated HCC. Data from various studies reveal that the co-ordinated actions of genetic instability, impaired lipid metabolism, increased oxidative stress altered lipid metabolism, hepatocyte apoptosis, inflammation, fibrosis and altered hormone signalling contribute to the development of HCC. These pathways likely act simultaneously and in combination to activate genetic and epigenetic mechanisms that cause progression of NAFLD and promote the development of NAFLD/NASH-associated HCC. At the clinical level, currently, it is not possible to determine which patients with NASH are most prone to develop HCC. Further studies are required to identify the patients who are at a risk of developing HCC. The identification of specific biomarkers is essential for predicting the transition from NASH to HCC. Currently, there are no pharmacological therapies for the prevention or treatment of NASH and NASH-associated HCC, thus understanding the mechanisms for the pathogenesis of these conditions may lead to the development of novel therapies. Anti-fibrotic, anti-diabetic, anti-inflammatory, antibiotics/probiotics and lipid-lowering drugs either alone or in combination could hold promise for the treatment for NAFLD/NASH-associated HCC.
